# MRI-based radiomics for prognosis of pediatric diffuse intrinsic pontine glioma: an international study

**DOI:** 10.1093/noajnl/vdab042

**Published:** 2021-03-05

**Authors:** Lydia T Tam, Kristen W Yeom, Jason N Wright, Alok Jaju, Alireza Radmanesh, Michelle Han, Sebastian Toescu, Maryam Maleki, Eric Chen, Andrew Campion, Hollie A Lai, Azam A Eghbal, Ozgur Oztekin, Kshitij Mankad, Darren Hargrave, Thomas S Jacques, Robert Goetti, Robert M Lober, Samuel H Cheshier, Sandy Napel, Mourad Said, Kristian Aquilina, Chang Y Ho, Michelle Monje, Nicholas A Vitanza, Sarah A Mattonen

**Affiliations:** 1 Stanford University School of Medicine, Stanford, California, USA; 2 Department of Radiology, Lucile Packard Children’s Hospital, Stanford University School of Medicine, Stanford, California, USA; 3 Department of Radiology, University of Pittsburgh Medical Center, Pittsburgh, Pennsylvania, USA; 4 Department of Radiology, Seattle Children’s Hospital, Seattle, Washington, USA; 5 Harborview Medical Center, Seattle, Washington, USA; 6 Department of Medical Imaging, Ann and Robert H. Lurie Children’s Hospital of Chicago, Chicago, Illinois, USA; 7 Department of Radiology, New York University Grossman School of Medicine, New York, New York, USA; 8 University College London, Great Ormond Street Institute of Child Health, London, UK; 9 Departments of Clinical Radiology & Imaging Sciences, Riley Children’s Hospital, Indiana University, Indianapolis, Indiana, USA; 10 Department of Radiology, CHOC Children’s Hospital, Orange, California, USA; 11 University of California, Irvine, California, USA; 12 Department of Neuroradiology, Bakircay University, Cigli Education and Research Hospital, Izmir, Turkey; 13 Department of Neuroradiology, Health Science University, Tepecik Education and Research Hospital, Izmir, Turkey; 14 Department of Radiology, Great Ormond Street Hospital for Children, London, UK; 15 Department of Medical Imaging, The Children’s Hospital at Westmead, The University of Sydney, Westmead, Australia; 16 Department of Neurosurgery, Dayton Children’s Hospital, Wright State University Boonshoft School of Medicine, Dayton, Ohio, USA; 17 Department of Neurosurgery, University of Utah School of Medicine, Salt Lake City, Utah, USA; 18 Department of Radiology, Stanford University, Stanford, California, USA; 19 Radiology Department Centre International Carthage Médicale, Monastir, Tunisia; 20 Department of Neurology and Neurological Sciences, Stanford University, Stanford, California, USA; 21 Division of Pediatric Hematology/Oncology, Department of Pediatrics, Seattle Children’s Hospital, Seattle, Washington, USA; 22 Ben Towne Center for Childhood Cancer Research, Seattle Children’s Research Institute, Seattle, Washington, USA; 23 Department of Medical Biophysics, Western University, London, Onatrio, Canada; 24 Department of Oncology, Western University, London, Ontario, Canada

**Keywords:** diffuse intrinsic pontine gliomas, diffuse midline glioma, H3K27M-mutant, machine learning, magnetic resonance imaging, radiomics

## Abstract

**Background:**

Diffuse intrinsic pontine gliomas (DIPGs) are lethal pediatric brain tumors. Presently, MRI is the mainstay of disease diagnosis and surveillance. We identify clinically significant computational features from MRI and create a prognostic machine learning model.

**Methods:**

We isolated tumor volumes of T1-post-contrast (T1) and T2-weighted (T2) MRIs from 177 treatment-naïve DIPG patients from an international cohort for model training and testing. The Quantitative Image Feature Pipeline and PyRadiomics was used for feature extraction. Ten-fold cross-validation of least absolute shrinkage and selection operator Cox regression selected optimal features to predict overall survival in the training dataset and tested in the independent testing dataset. We analyzed model performance using clinical variables (age at diagnosis and sex) only, radiomics only, and radiomics plus clinical variables.

**Results:**

All selected features were intensity and texture-based on the wavelet-filtered images (3 T1 gray-level co-occurrence matrix (GLCM) texture features, T2 GLCM texture feature, and T2 first-order mean). This multivariable Cox model demonstrated a concordance of 0.68 (95% CI: 0.61–0.74) in the training dataset, significantly outperforming the clinical-only model (*C* = 0.57 [95% CI: 0.49–0.64]). Adding clinical features to radiomics slightly improved performance (*C* = 0.70 [95% CI: 0.64–0.77]). The combined radiomics and clinical model was validated in the independent testing dataset (*C* = 0.59 [95% CI: 0.51–0.67], Noether’s test *P* = .02).

**Conclusions:**

In this international study, we demonstrate the use of radiomic signatures to create a machine learning model for DIPG prognostication. Standardized, quantitative approaches that objectively measure DIPG changes, including computational MRI evaluation, could offer new approaches to assessing tumor phenotype and serve a future role for optimizing clinical trial eligibility and tumor surveillance.

**Key Points**

- This is the first discovery-driven, 3D MRI machine learning study to prognosticate DIPG.
- Radiomics-based heterogeneous tumor intensity and texture features on MRI confer a better prognosis.
- We identify features that are significant and preserved across multiple institutions to examine clinical applicability.

**Importance of the Study**

With new clinical trials underway for DIPG, including immune-based therapies, there is a need for quantitative prognostic biomarkers that can more objectively assess tumor risk factors and assist clinical trial eligibility and therapy planning. In this international study, we highlight the potential use of radiomics and machine learning to better prognosticate outcomes for patients with DIPG than clinical variables alone. Our pilot results highlight the potential for radiomics and machine learning to contribute to precision in neuro-oncology and potentially augment clinical decision making for this devastating group of tumors.

Diffuse intrinsic pontine gliomas (DIPGs) are lethal brain tumors that predominantly affect children. With a median survival of 11 months, the prognosis remains dismal.^1,2^ The presence of the *H3 K27M* mutation in approximately 80% of patients has led to the World Health Organization (WHO) classification of “diffuse midline glioma, H3 K27M-mutant” (DMG).^3,4^ However, the term “DIPG” remains clinically relevant given the unique clinical characteristics of pontine DMG, the need to include H3 wildtype tumors in the definition, and the practical consideration that *H3 K27M* status may be unknown for many unbiopsied tumors. At present, MRI is the mainstay for tumor diagnosis, evaluation of tumor extent, presurgical biopsy planning, and therapy response.^5^

Previous studies have reported that MRI features such as tumor size or contrast enhancement correlate with tumor progression, radiation effects, and/or tumor necrosis.^6–8^ Studies have also applied different MRI techniques to describe tumor physiology,^5,9^ chemical signatures,^10^ or tissue microstructure^11,12^ that might confer prognostic information. However, clinical translatability of these imaging tools may be limited by differences in imaging techniques and protocols, as well as the labor and cost of image post-processing.

With current and new clinical trials underway for DIPG, including immune-based therapies, there is a need for noninvasive prognostic imaging biomarkers that can more precisely stratify tumor risk factors and thereby assist clinical trial eligibility and therapy planning. A recent International DIPG Registry study of 357 patients reported that age and distant disease best predicted length of survival, while there was excessive discordance among expert human readers regarding MRI features.^9^

Advances in computer vision have shown potential for image-based oncologic evaluation, including machine learning for prognostic modeling.^13–17^ While human visual inspection offers information regarding macroscopic tumor environment such as tumor location, size, contrast enhancement, hemorrhage, or diffusion changes, computational approaches could uncover clinically significant high-dimensional image features that elude visual inspection. Radiomics algorithms can extract mineable high-dimensional, quantitative image features, which can then be used to create machine learning models predictive of clinical outcomes.^18–20^ Standardized analyses allow clinician investigators to test the reproducibility and replicability of these models.^21^ Furthermore, a machine-based quantitative model that generalizes, despite *heterogeneous* data acquired from different centers, could reduce human interobserver differences even among experts. For this purpose, we assembled an international cohort of children with DIPG tumors and investigated radiomics approaches to identify important computational features for DIPG prognosis.

## Materials and Methods

### Study Cohort

For this multicenter, retrospective study, institutional review board approval was obtained at all participating institutions. Stanford Children’s Hospital served as the host institution and data use agreements were obtained at all participating sites: Seattle Children’s Hospital (SC—Seattle, Washington), Primary Children’s Hospital (UT—Salt Lake City, Utah), Children’s Hospital Orange County (CH—Orange County, California), Dayton Children’s Hospital (DY—Dayton, Ohio), Indiana University Riley Hospital for Children (IN—Indianapolis, Indiana), Great Ormand Street Hospital (GO—London, United Kingdom), Centre International Carthage Médical (TM—Monastir, Tunisia), Lurie Children’s Hospital of Chicago (CG—Chicago, Illinois), NYU Langone Medical Center (NY—New York City, New York), and Tepecik Health Sciences (TK—Izmir, Turkey). We included all DIPG patients younger than 19 years old with a baseline brain MRI prior to therapy. Diagnosis was made based on MRI evaluation and consensus between 2 neuroradiology faculty. Exclusion criteria were nondiagnostic MRI due to motion or extensive metal artifacts, lack of follow-up information, patients who refused standard radiotherapy, and given that distant disease outside of brainstem is a known predictor of poor outcome,^9^ patients known to have metastatic disease at presentation. Patients who were alive were censored at the time of the last known follow-up.

### MRI Acquisition

MRI brain scans were acquired at either 1.5 and 3 T magnet using the following vendors across the centers: GE Healthcare, Siemens AG, Philips Healthcare, and Toshiba Canon Medical Systems USA Inc. The T2-weighted MRI (T2-MRI) scans were T2 TSE clear/sense, T2 FSE, T2 propeller, T2 blade, T2 drive sense (TR/TE 2475.6-9622.24/80-146.048); slice thickness 1–5 mm with 0.5 or 1 mm skip; matrix ranges of 224-1024 × 256-1024. T1-weighted post-gadolinium MRI (T1-MRI) scans included T1 MPRAGE, T1 BRAVO, T1 FSPGR, T1 SPGR, and T1 SE; slice thickness 0.8–1.2 mm; matrix (256-512) × (256-512). To ensure consistency across sites, all images were collected in DICOM image format.

### Tumor Volume

**Figure 1.**
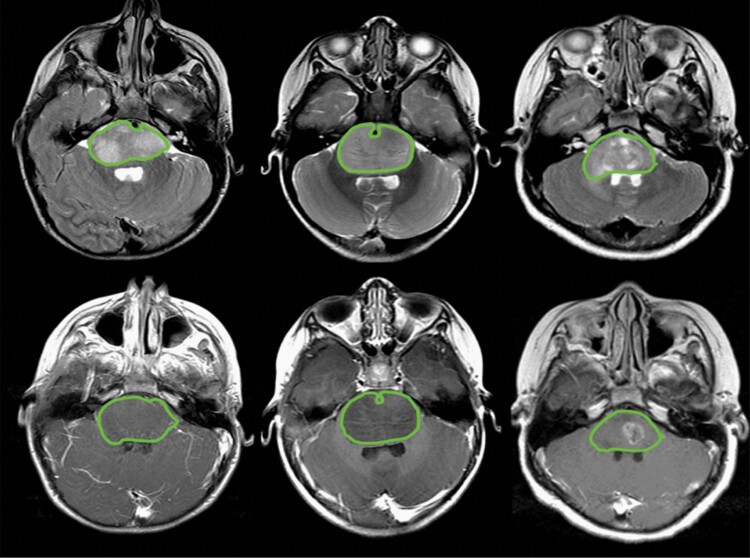
Examples of tumor regions of interest over the tumor. Tumor segmentation was performed over abnormal signals on T2-weighted MRI (top) and corresponding gadolinium-enhanced T1-weighted MRI (bottom) to generate tumor volume.

Manual delineation of tumor boundaries was performed independently on T2-MRI and over the corresponding tumor boundary on T1-MRI regardless of enhancement using *Osirix* software (Switzerland; Figure 1). The tumor boundary was determined by consensus review among experts (K.W.Y., R.M.L., S.H.C.). Segmentation output served to generate tumor volumes, which were then used for radiomics feature extraction.

### Radiomics Feature Extraction

Radiomic features were extracted from within the segmented tumor boundaries using the open-source *PyRadiomics* software (version 2.2.0.post7+gac7458e) using the configuration file shown in Supplementary Appendix S1 and implemented in the Quantitative Image Feature Pipeline.^21,22^ Prior to feature extraction, images were normalized (normalize scale = 100) and resampled to isotropic 1 mm voxels. A total of 900 features were extracted on each T2-MRI and T1-MRI. Extracted features included size, shape, first-order, and texture-based features computed on original, wavelet, and Laplacian of Gaussian filtered images. Wavelet images were filtered with a high band-pass (H) or low band-pass filter (L) in the x, y, and z directions resulting in 8 different combinations of decompositions. A bin width of 10 was used for gray-level discretization in both normalized MR images.

### Model Development

The complete dataset was randomly divided into training (60%, *n* = 106) and test (40%, *n* = 71) sets. The training set was used to select the optimal features to predict overall survival (OS) and build the Cox regression model. The model was then locked and evaluated on the holdout test set. All radiomic features were standardized prior to model development. We evaluated the utility of clinical features alone (age at diagnosis and sex), radiomic features alone, and the combination of clinical and radiomic features. All model development and evaluation were performed using RStudio (version 1.3.959)^23^ with a priori statistical significance of α less than 0.05.

The *glmnet* package (version 4.0-2)^24^ was used to select the most important features to predict OS in the training dataset. We performed 100 repetitions of 10-fold cross-validation to fit a Cox regression model based on the least absolute shrinkage and selection operator regularization (α = 1). The optimal features were chosen based on the lambda value with the minimum cross-validated error across the 100 repetitions. The optimal features were then used to build a Cox proportional hazards model in the training dataset. This model was then locked and evaluated in the testing dataset using the concordance index. Noether’s test was used to determine the significance of the concordance index compared to random chance (concordance = 0.5). The concordance indices of different models were compared using the Student’s *t*-test for dependent samples in the *survcomp* package (version 1.34.0).^25^ We also performed Kaplan–Meier analyses to risk-stratify patients based on the median risk score from the Cox model in the training set, with significance determined by the log-rank test.

## Results

### Demographics and Clinical Information

One hundred seventy-seven patients (85 males, median age 6.7 [range 1.6–19] years) were included for analysis. OS was available for 147 patients (median survival 11 [range 1–74] months). Thirty patients were alive at the time of analysis (median follow-up of 6 [range 1–164]). Clinical information including patient demographics is summarized in Table 1. While all patients received standard focal radiation therapy of approximately 54 Gy, therapy after radiation varied from no further therapy, to an early-phase clinical trial, to a conventional chemotherapy regimen.^26,27^

**Table 1.** Patient Demographics

Sex	*n* (% of total)
Male	85 (48)
Female	92 (52)
Total	177
Age	Average (range), months
	80 (19–229)
Institution	*n* (% of total)
CG	10 (6)
CH	4 (2)
DY	5 (3)
GO	12 (7)
IN	19 (11)
NY	13 (7)
SC	37 (21)
ST	60 (34)
TK	4 (2)
TU	2 (1)
UT	11 (6)
Imaging	*n* (% of total)
T1 only	18 (10)
T2 only	6 (3)
T1 and T2	153 (86)
Overall survival^a^	Average (range), months
	11 (11–164)

^**a**^Overall survival is calculated from the date of diagnosis and date of death or the last known follow-up.

### MRI-Based Radiomics

#### Model development

Since T1- and T2-MRI are standard of care imaging, we sought to build an initial model using both sets of radiomic features. Since not all patients in our dataset had both T1- and T2-MRI available for analysis, we built the model on the 95 patients (90%) in the training dataset which had radiomic features on both T1- and T2-MRI. Image examples from these patients are shown in Supplementary Figure S1. This training dataset was used to perform feature selection. The lambda value with the minimum cross-validated error across the 100 repetitions resulted in a total of 5 features with non-zero coefficients. This included 3 features from T1-MRI and 2 features from T2-MRI. All features were intensity and texture-based on the wavelet-filtered images. The T1-MRI features included wavelet (LLH) gray-level co-occurrence matrix (GLCM) inverse difference normalized (IDN), wavelet (LHH) GLCM informational measure of correlation 2 (IMC2), and wavelet (HHH) GLCM IMC2. The T2-MRI features were wavelet (LLH) GLCM IDN and wavelet (HHH) first-order mean.

**Figure 2.**
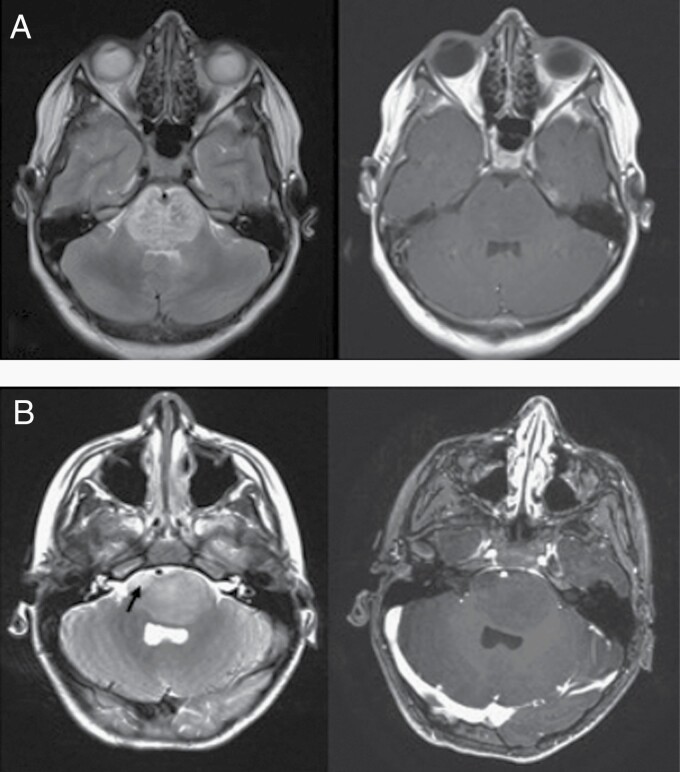
A visual example of MRI radiomics. Example axial T2 (left) and T1 (right) MRI for 2 children. T2-weighted MR images of a patient who survived 20 months (A) and a patient who survived only 3 months (B) show heterogeneous or coarse intensity distribution with punctate foci of dark signal interspersed within T2 hyperintensities in the patient (A) compared to (B), where more confluent intensities are seen with a more localized T2 hyperintense soft tissue abnormality in right anterior pons (arrow). The T1 post-contrast MRI demonstrates limited qualitative characteristics of the tumor.

This multivariable Cox model demonstrated a concordance of 0.68 (95% CI: 0.61–0.74) in the training dataset. This model outperformed the clinical model that used sex and age at diagnosis as variables, which had a concordance of 0.57 (95% CI: 0.49–0.64), *P* = .017. When clinical features were combined with radiomics, the model performance increased to a concordance of 0.70 (95% CI: 0.64–0.77), but this was not an improvement over radiomic features alone (*P* = .05). Neither clinical feature contributed significantly to the model (Table 2) nor was there any correlation between the clinical or radiomic features, as shown in Supplementary Figure S2. A qualitative example of imaging characteristics seen in 2 patients is shown in Figure 2.

**Table 2.** Multivariable Cox Model for Combined Radiomic and Clinical Features Model

Feature	Hazard Ratio (95% CI)	*P*
T1 wavelet (LLH) GLCM IDN	1.31 (0.99–1.73)	.06
T1 wavelet (LHH) GLCM IMC2	0.97 (0.72–1.30)	.83
T1 wavelet (HHH) GLCM IMC2	0.68 (0.50–0.92)	.01*
T2 wavelet (LLH) GLCM IDN	1.33 (1.04–1.71)	.02*
T2 wavelet (HHH) first-order mean	1.36 (1.02–1.82)	.04*
Sex	1.47 (0.90–2.41)	.13
Age	1.00 (1.00–1.01)	.25

*Indicates statistical significance (ie, p < .05)

#### Independent evaluation

The Cox proportional hazard model using both T1 and T2 features was locked based on the training dataset and independently evaluated in the holdout test set (*n* = 58). During testing, the clinical-only (0.51 [95% CI: 0.42–0.59]) and radiomics-only models (0.55 [95% CI: 0.48–0.62]) performed similarly (*P* = .21).

**Figure 3.**
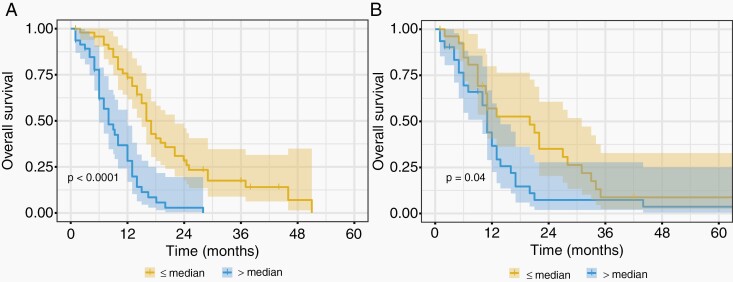
Kaplan–Meier curves for the Cox regression model including radiomics and clinical features in (A) the training dataset (*n* = 95, log-rank *P* < .0001) and (B) the testing dataset (*n* = 58, log-rank *P* = .04). Patients were stratified on the basis of the median risk value in the training dataset and the shaded regions represent the 95% confidence intervals.

In comparison, the combined radiomics and clinical model was validated in the independent testing dataset (0.59 [95% CI: 0.51–0.67], Noether’s test *P* = .02). In the independent test dataset, the combined model outperformed the clinical-only (*P* = .04) and the radiomics-only (*P* = .003) models. The combined model also risk-stratified patients based on the median risk score determined in the training dataset (Figure 3). A summary of all concordance indices is shown in Table 3.

**Table 3.** Concordance (95% CI) Metrics for All Models Using Both T1 and T2 MRI Features in the Training and Testing Datasets

Model	Training (*n* = 95)	Testing (*n* = 58)
Clinical features	0.57 (0.49–0.64)	0.51 (0.42–0.59)
Radiomic features	0.68 (0.61–0.74)^a^	0.55 (0.48–0.62)
Clinical + Radiomic features	0.70 (0.64–0.77)^a^	0.59 (0.51–0.67)^a^

^a^Indicates significance based on Noether’s test to determine significance from random (concordance = 0.5).

#### Performance of individual T1 contrast and T2 features

To investigate the utility of T1 and T2 imaging features alone, we evaluated the performance of the three T1 features on all patients with gadolinium-enhanced T1-MRI available. Likewise, the two T2 features were evaluated on all patients who had T2-MRI available. The results are given in Supplementary Table S1. The performance of individual sequences was lower compared to the combination of T1 and T2 MRI radiomic features, and none of the models were validated in the testing dataset.

## Discussion

Using high-dimensional feature analysis of a large pooled international MRI dataset of DIPG patients, a combined model incorporating clinical variables (age and sex) and radiomics features was able to predict OS better than clinical variables alone. While DIPG remains highly lethal, age is a known prognostic factor for DIPG, with longer survival reported for older children.^6^ Based on radiologist qualitative and quantitative evaluations of variable imaging features, several studies have reported that various baseline MRI features correlate with survival, but with inconsistent associations.^6,8,28–31^ This likely relates to small cohort sizes, heterogeneous imaging protocols, human interobserver differences, and differences in criteria used for tumor descriptors. In a recent study of 357 pediatric DIPG, image features, such as extrapontine tumor, larger size, enhancement, necrosis, reduced diffusion, and distant (outside of the brainstem) tumor spread correlated with shorter OS in a univariate analysis. However, only age and distant disease were significant predictors in multivariate analysis.^9^ Importantly, due to significant discordance among human readers—depending on the specific image features analyzed—the study recommended a central review or consensus expert opinion if clinical trials are considered.

Machine learning has shown success in uncovering clinically significant high-dimensional image features that could assist precision in oncologic evaluation.^13–17^ Using high-throughput feature extraction from tumor image volume, radiomics enables the *mining* of quantitative image features relevant to tumor diagnosis, prognosis, or genomics.^32,33^ In the evaluation of adult glioblastoma, for example, studies have identified several significant computational image features predictive of clinical outcomes and underlying genomic signatures.^34–37^ In children, machine learning has shown promise in characterizing different tumor pathologies using texture-features^38^; for example, in medulloblastoma, tumor edge sharpness features were found to correlate with Sonic Hedgehog or Group 4 tumors.^39^

In this study, we developed an MRI-based machine learning model predictive of OS, which performs better than clinical variables alone, such as age or sex. We used the publicly available, open-source PyRadiomics^21^ approach to compute features and thereby enable future reproducibility and replicability of feature extraction. To capture diversity in data that relate to imaging protocols and vendor-dependent MRI hardware, and to develop a model that is generalizable across centers, we pooled data across international sites. The *radiomics-only* model outperformed clinical features of age or sex in our training cohort. On an *independent* test dataset, combined radiomics and clinical model outperformed the clinical and radiomics models alone and was able to risk-stratify patients based on the median risk score determined in the training dataset.

To our knowledge, this study represents the first MRI-based *machine learning* approach for DIPG prognosis. Using a software for MRI-based texture analysis on a single 2D image slice of DIPG tumors, one study of 32 patients suggested “homogeneous” texture may have worse outcome.^40^ In this study, we conducted a 3D image analysis and applied a *discovery-driven* approach in search of significant computational features to create a prognostic machine learning model. Combined gadolinium-enhanced T1-MRI and T2-MRI outperformed either MRI sequence alone. Of the 900 features extracted on each image series, feature selection identified 5 intensity- and texture-based features from *wavelet-filtered images*. Applying a filter to the image prior to calculating radiomic features allows for identifying patterns or highlighting additional details within the image, including both fine and coarse textures. A wavelet filter therefore allows us to further enhance textures that might be present in an image but difficult to appreciate with the human eye. Many of the features also represented GLCM quantitative features that compute different pixel combinations of gray levels within a tumor volume.

Although they are difficult to translate using traditional image descriptors and radiology lexicon, our results suggest that *heterogeneous tumor pixel intensity* or *texture* confer a better prognosis. For example, tumors with more heterogenous gray-level distribution (or high T2 wavelet [LLH] GLCM IDN, T1 wavelet [LLH] GLCM IDN, T1 wavelet [LHH] GLCM IMC2 scores) or more complex texture (T1 wavelet [HHH] GLCM IMC2) were found to have longer survival. There were no size or shape features selected in this study, suggesting the importance of image *texture* appearance—beyond that which can be appreciated by the human eye in predicting prognosis. It is worth noting that first-order features on the original T1 contrast-enhanced image were calculated and would be reflective of any enhancement that may be present within the tumor. Similarly, the volume and diameter of the tumor were also included as radiomic features; however, none of these were selected for the final model. Although underlying biologic correlate of T2 heterogeneous intensities remains to be investigated. It is possible these regions reflect regions of heterogeneous tumor cell density, heterogeneous immune cell infiltrates, heterogeneity of extracellular matrix composition, and/or variable vasculature proliferation. Future postmortem histological studies would be needed to further clarify the histological correlates to these imaging findings.

DIPG is currently treated with radiotherapy, which provides temporary stabilization of symptoms and extends OS by an average of 3 months.^41^ Unfortunately, over time, the tumor inevitably progresses. Using machine learning approaches, we describe computationally derived prognostic image features from routine MRI, such as T2- and gadolinium-enhanced T1-weighted imaging. More sophisticated methods for image analytics have never been more important due to both current treatments and the next wave of clinical trials.^42^ Many patients are dosed with bevacizumab that affects local edema^26,43,44^ and often complicates assessments of tumor progression. Considering the failure of hundreds of chemotherapy regimens,^45^ a new wave of immunotherapy clinical trials ranges from immunotherapy options such as IDO inhibitors (NCT02502708), vaccines (NCT02960230), or CAR T cells (NCT04185038 and NCT04196413).

Despite iRANO working group guidelines for monitoring CNS tumor immunotherapy patients,^46^ patients receiving immunotherapy regularly face critical moments of distinguishing true progression from pseudo-progression.^47–50^ Standardized, quantitative approaches to neuroimaging evaluation that objectively measures DIPG changes, including computational-based evaluation using digital tumor image data, could contribute to optimizing these trials and potentially offer new approaches to evaluating changes in tumor phenotype that relate to different therapies.

Our study was retrospective in nature and involved multiple institutions including international centers. Inevitably, there was heterogeneity in imaging protocols (eg, 1.5 vs 3.0 T) and scanner vendors. However, by using multicenter data, we sought to identify prognostic features that were *preserved* despite heterogeneity in image data, such as gray-scale contrast, dynamic range, intensity values, and others, and were therefore generalizable across centers. We did not investigate diffusion tensor or perfusion MRI, but specifically focused on routine conventional MRI. If biomarkers identifiable on routine MRI were found clinically significant despite heterogeneity in imaging protocols, the ubiquity of these features could more easily facilitate clinical translation across centers. Stratification by treatment was not possible due to the large sample size and heterogeneous clinical centers, which is a limitation in our findings. Although therapy after radiation varied, all patients received standard radiotherapy approximately 54 Gy to the brainstem. Future studies may warrant the consideration of prospectively evaluating these radiographic parameters in patients with uniform treatment. Incorporation of parameters may be critical as patient numbers are small and outliers in survival can significantly alter the interpretation of study results. With a more systematic, stratified review of exceptional responders, such an approach would allow a more critical review of their course and evaluate if they may inherently have had a less aggressive form of the disease.

Using conventional MRI, we identified prognostic features from *wavelet-filtered* images, *which would not* be visible to the human eye, highlighting a unique role for computational-based feature analysis. We evaluated manual delineations of the regions of interest based on consensus review. However, tumor segmentations may still be subject to inter- and intraobserver variability. Future studies should evaluate the impact of the radiomic features on segmentation variability and semiautomated methods for tumor volume extraction should be investigated. Although we had an independent testing dataset, it was relatively small; and this model should be validated on a larger dataset, ideally with uniform treatment.

While molecular subgroups do have small but statistically significant differences in survival, in practical clinical terms all DIPGs carry the same fatal prognosis and there is no consensus on the degree of granularity in sub-classifying these tumors, as beyond mutations in the genes encoding histone 3, other mutations can occur in other significant genes.^4^ Furthermore, preclinical studies have often found that even epigenetically targeted agents such as HDAC inhibitors are still efficacious against histone wildtype DIPG, supporting some inherent similarities despite molecular distinctions.^51,52^ While, it would be desirable to identify radiomic features unique to the H3 K27M-mutant subgroup, this was not feasible due to lack of biopsy in the majority of the cases. As biopsy becomes more common, prospective associations between artificial intelligence-based imaging and genomics may provide further insight. However, as the majority of children worldwide with DIPG do not undergo biopsy and, therefore, cannot be classified as DMG as per the WHO classification, there remains an inherent benefit in studies such as this that evaluate all patients with “DIPG.” Despite these limitations, we demonstrate the potential of an MRI-based machine learning approach to DIPG prognostication. When used in conjunction with current clinical diagnostic methods, radiomics can be a noninvasive way to stratify tumors and predict survival using readily available, standard of care MR images.

## Conclusions

In this multi-institutional study, we highlight the potential of using radiomics and machine learning to better prognosticate outcomes for patients with DIPG than clinical variables alone. Imaging-based radiomic signatures could be used as a noninvasive biomarker that could potentially augment the clinical management and decision making for this devastating group of tumors.

## Supplementary Material

vdab042_suppl_Supplementary_Material

## Notes

**Authorship Statement.** J.N.W., A.J., A.R., S.T., Ma.M., E.C., A.C., H.A.L., A.A.E., O.O., K.M., D.H., T.S.J., R.G., R.M.L., S.H.C., S.N., M.S., K.A., C.Y.H., Mi.M., and N.A.V. collected, analyzed, and provided data from their respective institutions. L.T.T. and K.W.Y. organized, collated, and analyzed data from all centers. M.H. and S.A.M. built the model. L.T.T., K.W.Y., N.A.V., and S.A.M. conceived the project and wrote the manuscript. All authors contributed to editing the manuscript and approved the final version. K.W.Y., S.A.M., and N.A.V. supervised all aspects of the work.
