# Supplementary material for: MRI-based radiomics for prognosis of pediatric diffuse intrinsic pontine glioma: an international study
Source: Neurooncol Adv. 2021 Mar 5;3(1):vdab042. doi: 10.1093/noajnl/vdab042 (PMC8095337; doi:10.1093/noajnl/vdab042)
Supplement: vdab042_suppl_Supplementary_Material [file vdab042_suppl_supplementary_material.docx]

**SUPPLEMENTAL MATERIAL**

**Appendix S1**

setting:

normalize: true

normalizeScale: 100

binWidth: 10

label: 1

interpolator: 'sitkBSpline' # This is an enumerated value, here None is not allowed

resampledPixelSpacing: [1,1,1] # This disables resampling, as it is interpreted as None, to enable it, specify spacing in x, y, z as [x, y , z]

weightingNorm: # If no value is specified, it is interpreted as None

geometryTolerance: 0.0001

correctMask: True

imageType:

Original: {} # for dictionaries / mappings, None values are not allowed, '{}' is interpreted as an empty dictionary

LoG: {'sigma': [5,3,1]}

Wavelet: {}

featureClass:

shape: ['VoxelVolume',

'MeshVolume',

'SurfaceArea',

'SurfaceVolumeRatio',

'Sphericity',

'SphericalDisproportion',

'Maximum3DDiameter',

'Maximum2DDiameterSlice',

'Maximum2DDiameterColumn',

'Maximum2DDiameterRow',

'Elongation',

'Flatness'] # Only enable these shape descriptors (disables redundant Compactness 1 and Compactness 2)

firstorder: [] # specifying an empty list has the same effect as specifying nothing.

glcm: # for lists none values are allowed, in this case, all features are enabled

glrlm:

glszm:

**Table S1:** Concordance [95% CI] metrics for all models using only T1 or T2 MRI features in the training and testing datasets.

| **Model** |  | **T1 MRI Only** | |  | **T2 MRI Only** | |
| --- | --- | --- | --- | --- | --- | --- |
|  |  | **Training (n=99)** | **Testing (n=60)** |  | **Training (n=102)** | **Testing(n=69)** |
| Radiomics |  | 0.63 [0.55-0.70]* | 0.51 [0.44-0.58] |  | 0.62 [0.56-0.69]* | 0.55 [0.48-0.62] |
|  |  |  |  |  |  |  |
| Clinical + Radiomics |  | 0.66 [0.59-0.73]* | 0.56 [0.49-0.63] |  | 0.64 [0.57-0.71]* | 0.56 [0.49-0.63] |


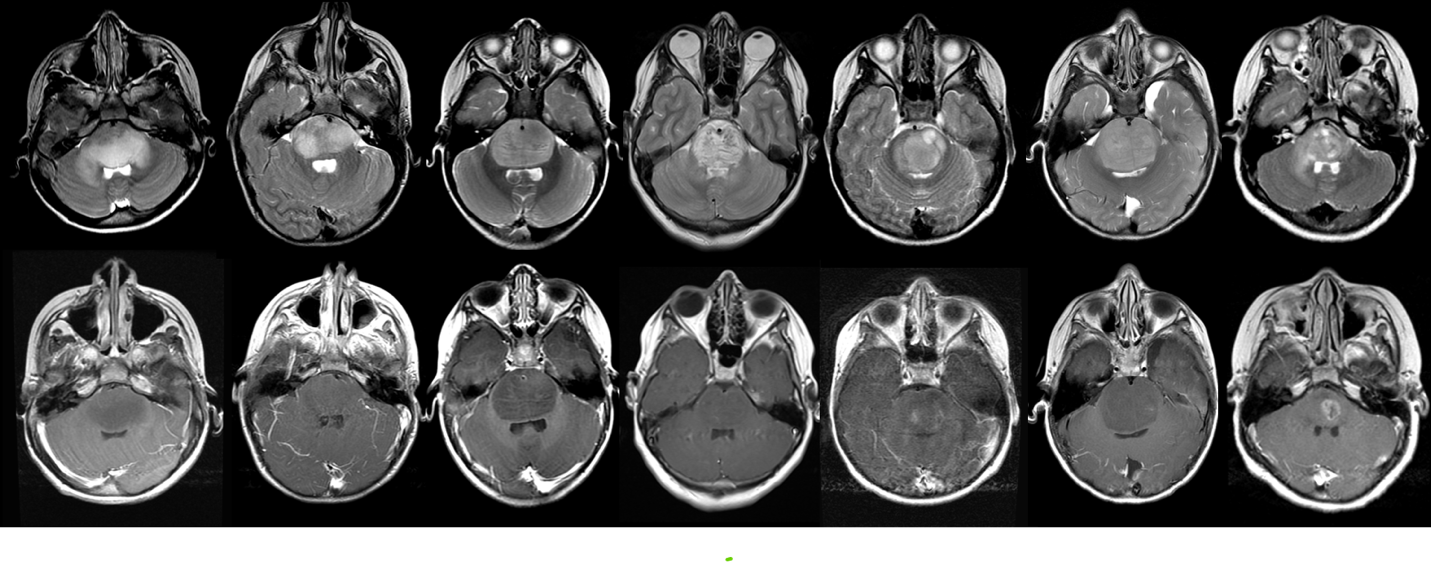


**Figure S1.** **Various image features of DIPG at diagnosis.** T2-weighted (top) and corresponding gadolinium-enhanced T1 weighted (bottom) MRI are shown for each patient. Note diverse range of T2 signal, including intensity range (T2 bright, isointense, and dark regions), and distribution of intensities (homogeneously bright or patchy bright or dark) within the tumor. While many tumors are non-enhancing, some demonstrate patchy or irregular enhancement. Tumor volume can be isolated to extract high-dimensional radiomics features that might relate to tumor prognosis

**
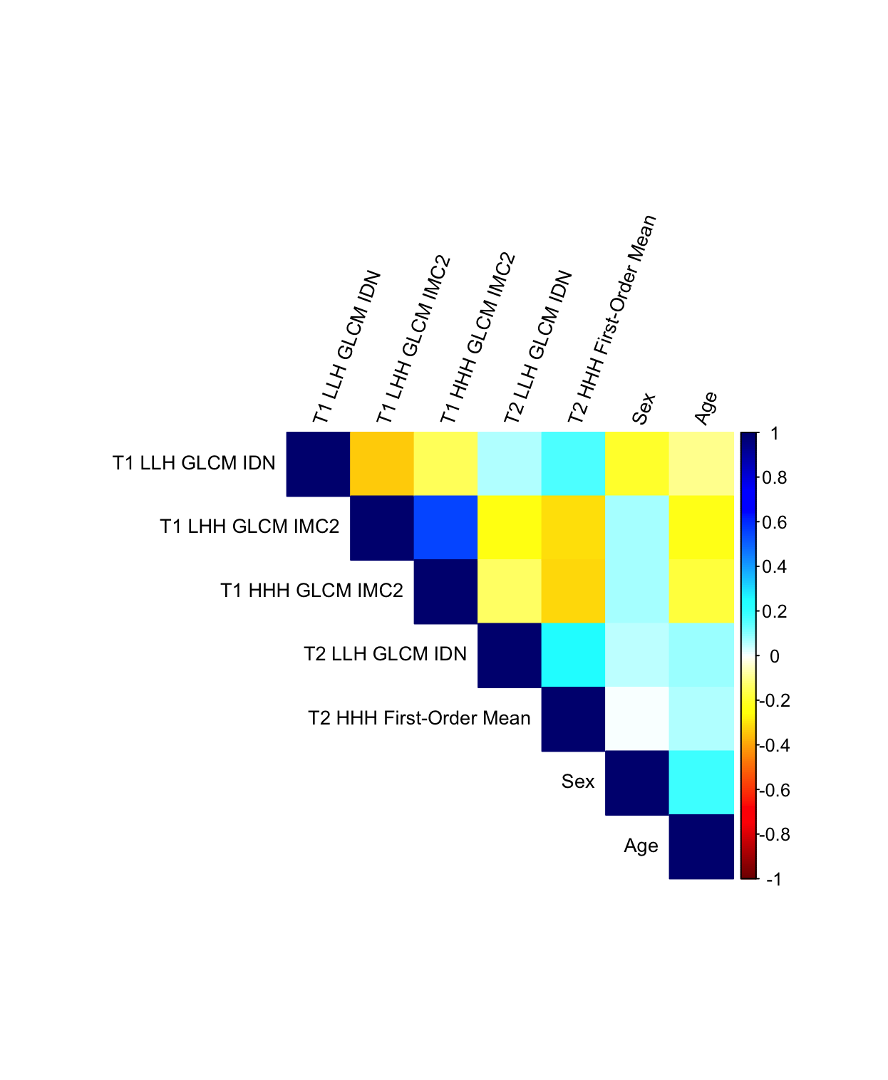
**

**Figure S2: Pearson correlation heatmap for all clinical and selected radiomics.** There was no significant correlation between any of the features.
